# Assessment of the neuromuscular characteristics of the flexo-extension muscles of knee by tensiomyography in elite soccer players: a systematic review

**DOI:** 10.3389/fspor.2025.1610094

**Published:** 2025-09-22

**Authors:** Jesús Lorigados Pérez, Álvaro Bustamante-Sánchez

**Affiliations:** Department of Sports Sciences, Faculty of Medicine, Health and Sports, Universidad Europea de Madrid, Madrid, Spain

**Keywords:** tensiomiography, knee, flexor-extensor, neuromuscular, soccer

## Abstract

**Introduction:**

The objective was to summarize the usefulness of tensiomyography (TMG) as a monitoring tool to evaluate the neuromuscular characteristics of the knee flexor and extensor muscles in elite male soccer players.

**Methods:**

A search of Pubmed, Web of Science (WOS), SPORTdiscus and Scopus databases was performed using the PRISMA methodology.

**Results:**

The results obtained suggest that TMG can provide information not only on individual responses to training, but also on neuromuscular and functional fatigue and lateral asymmetries.

**Discussion:**

The monitoring of the evolution of the neuromuscular state during the season in soccer players, can help coaches and medical staff to identify the initial characteristics that are relevant for the planning and programming of training, and that these changes can be monitored through the study of the modification of mechanical muscle characteristics assessed by tensiomyography.

**Conclusion:**

The TMG is a useful tool for evaluating the neuromuscular characteristics of the knee flexor and extensor muscles in elite male footballers.

## 1 Introduction

Soccer is an intermittent sport with alternating moments of high and low intensity, requiring players to have a high level of endurance and the ability to generate energy through the muscles of the lower limbs (1). The main specific qualities of physical fitness in soccer include explosiveness of the lower limbs, where the extensor and flexor muscles groups of the knee are involved in running, jumping and shooting, influencing stride length, stabilization of the knee joint when changing direction, acceleration and deceleration, and landing (1).

Neuromuscular capacities play an important role in contemporary soccer due to the progressive increase in the number of high-speed actions during matches (2). Given that the frequency of these events in modern football is on the rise due to a general intensification of the game, the frequency of situations involving a risk of possible hamstring injuries is also increasing (3). Situations where the player performs sprints, jumps or technical elements with the ball at maximum intensity, are considered to be the events with the greatest harmful production on the hamstring muscles (4, 5).

Tensiomyography (TMG) was developed as a method of muscle assessment at the Faculty of Electrical Engineering at the University of Ljubljana (Slovenia). In sport, it has been used as a valid and reliable tool for evaluating the contractile properties of skeletal muscle, obtaining valuable information on the effect of training loads on the muscular structures of athletes, based on its applicability, simplicity and innocuous effect (6).

Weekly training demands can alter muscle stiffness, with high levels before the training session being a discriminating factor for the incidence of injury (7, 8). The use of muscle stiffness measurements to evaluate neuromuscular function, can provide a more sensitive measure for the purpose of identifying athletic status (9).

TMG can provide information on muscle stiffness, contraction time, neuromuscular status after exercise, muscle tone and fatigue, as well as possible muscle imbalances and asymmetries (10, 11).

Similarly, TMG could be useful for the estimation of muscle fibre composition (type I for endurance, type II for strength and speed). This would facilitate the selection of athletes with a predisposition for specific sports (12).

Using a portable device, TMG can measure the individual surface properties of muscles by recording isometric contraction induced externally by an electro-stimulator (13), using an electrical stimulus of controlled intensity (11).

It has been found to be highly reliable for measuring the following variables and contractile parameters: maximum radial displacement (Dm), contraction time (Tc), response time (Td), relaxation time (Tr), and sustainment time (Ts), in the muscles of the lower limb vastus medialis (VM), vastus lateralis (VL), rectus femoris (RF) and biceps femoris (BF) (14–16).

The Dm corresponds to the maximum radial displacement of the muscle belly expressed in millimeters and is related to muscle stiffness. The Tc corresponds to the time elapsed between 10% and 90% of the Dm and has been related to the speed of force generation. The Td represents the time taken to reach 10% of the total displacement after stimulation and is related to the dominant fiber type, its state of fatigue and its level of potentiation and activation. Tr gives us information about fatigue levels. Ts indicates how long the contraction is maintained and is calculated by determining the time that has elapsed since the deformation has reached 50% of its maximum value, until it returns to 50% of the maximum deformation during relaxation (6) (Table 1).

**Table 1:** Description of TMG variables.

Variable	Description
Dm	Maximum radial displacement (mm). Indicates muscle stiffness.
Tc	Time between 10%–90% of Dm. Associated with force generation velocity.
Td	Time to reach 10% of Dm after stimulation. Reflects fibre type, fatigue, and activation status.
Tr	Provides information about fatigue levels.
Ts	Duration of contraction (50% deformation).
Vc	Contraction Velocity = Dm/(Tc + Td).

Mm, millimeters; Dm, maximum displacement; Tc, contraction time; Td, response time.

Finally, we can obtain a contraction velocity (Vc) parameter derived from the TMG, and it is calculated by dividing Dm by the sum of Tc and Td (1, 16, 17, 34) (Table 1).

Vc can be considered a very useful and applicable index due to its ability to detect functional changes in muscle mechanical properties, at least when these neuromuscular adaptations are related to players deficiencies in maximum sprint capacity and performance with respect to change of direction speed in professional soccer (1).

Parameters such as Tc, Td and Tr show a strong correlation with the proportion of type I fibres in muscle biopsies, accounting for up to 87% of the variance. This suggests that TMG may serve as a valuable and non-invasive alternative for estimating muscle fibre composition, thereby avoiding invasive techniques such as biopsy (12).

It is necessary to carry out physiological monitoring of players to evaluate the effectiveness of training based on knowledge of the player's initial state, since physical and physiological reference values are known to change as the season progresses (18).

The main objective of the review is to analyze the usefulness of the TMG device as a control tool to evaluate the neuromuscular characteristics of the knee flexor and extensor muscles in elite male soccer players.

## 2 Methods

### 2.1 Study design

**Figure 1:**
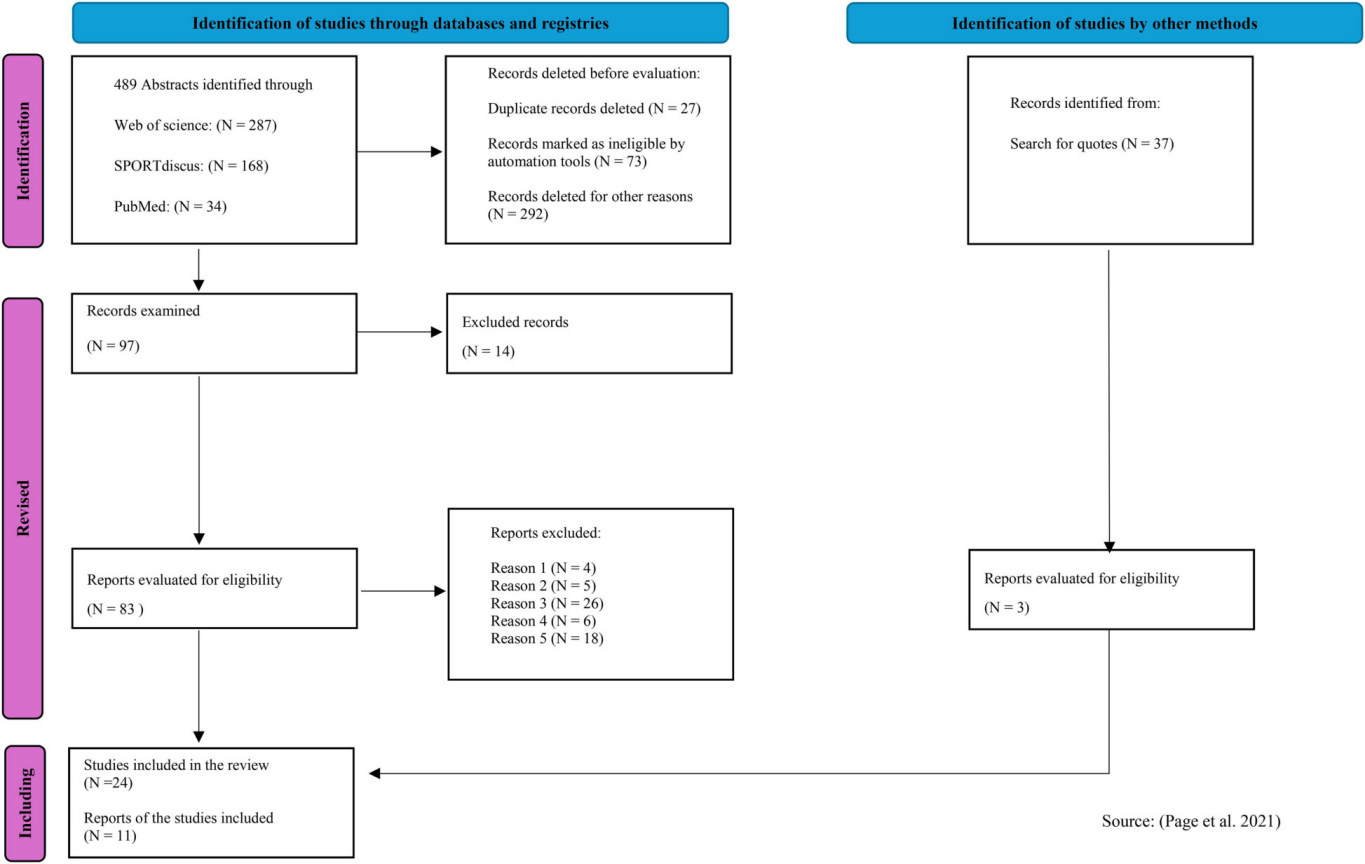
Flow chart. Source (19).

This systematic review has been prepared following the recommendations of the PRISMA (Preferred Reporting Items for Systematic Reviews and Meta-Analyses) statement of Page et al. (19) (Figure 1).

### 2.2 Search strategy

The search was carried out from January 1 to September 4, 2024, through the Dulce Chacón Library of the European University of Madrid in the Scopus, SPORTdiscus, WOS and PubMed Complete databases. The search was updated on September 30, 2024.

Pubmed Complete and WOS were chosen as they are currently the two most important databases in sports medicine and biomedicine respectively, providing a high detection rate of primary research (20). In addition, the recommendation of Prinsen et al. (21) to search for content-specific databases such as Sportdiscus and Scopus was followed.

The search was extended using reference lists of relevant articles to ensure the retrieval of as many related documents as possible.

Search filters were developed using a combination of keywords and term selection in the databases. MeSH (medical subject headings) terms were not used. The following keywords were used in combination using the Boolean operators AND/OR: Tensiomiography; Knee; Flexor-extensor; Neuromuscular; Soccer.

To avoid excluding relevant articles, methodological search filters were not used in the first instance (22).

The Cochrane database was consulted, and no results were found on TMG and soccer. The author also consulted experts in the field to include any additional relevant published or accepted studies.

### 2.3 Selection and admission criteria

Studies were selected according to the following inclusion criteria:

(1) The full text had to be available. (2) The studies had to be written in English. (3) They had to be strictly focused on the investigation of issues related to the neuromuscular characteristics of the knee flexor and extensor muscles measured by TMG. (4) Only articles published in scientific journals between January 2012 and August 2024. (5) The sample should be elite male soccer players.

The decision to include only elite male players in this systematic review was driven by the authors' intention to narrow the scope of the search as much as possible and thereby enhance the value of the analysis obtained. Similarly, studies published prior to 2012 were excluded due to the unavailability of modern TMG devices at that time.

The exclusion criteria were all those that were not included in the inclusion criteria.

A PRISMA 2020 flow diagram was created for new systematic reviews that include searches of databases, registries, and other sources, to illustrate the screening and selection process (Figure 1).

### 2.4 Data extraction

Firstly, the following data was extracted from the studies: (1) Author(s) and Year. (2) Objective(s). (3) Sample size and type. (4) Age, weight, height, body mass index (BMI): measure + standard deviation (SD). (5) Period of the season. (6) Muscle(s) measured. (7) Measured variables of TMG. A detailed listing and description of the extracted key parameters are shown in Table 2 and Table 3.

**Table 2:** Description of the studies.

Author(s), year	Objective	Sample	Age/Weight/Height	Season period
Rey et al. (2020) (23)	Analyse changes in muscle contractile properties during a weekly microcycle.	*N* = 19 professional players	26.0 ± 4.1 y; 77.5 ± 3.5 kg; 180.2 ± 4.2 cm	Competitive
Álvarez-Díaz et al. (2016) (24)	Compare dominant vs. non-dominant limb neuromuscular characteristics.	*N* = 38 male players (6 left-footed, 32 right-footed)	21.1 ± 4.9 y; 71.5 ± 10 kg; 175 ± 7 cm	Competitive
Fernández-Baeza and González-Millán (2020) (25)	Compare flexor-extensor properties in professionals vs. amateurs.	*N* = 38 (22 pros, 16 amateurs)	Pros: 26.3 ± 4.7 y; 73.4 ± 5.1 kg; 179.6 ± 5.6 cm/Amateurs: 21.1 ± 1.7 y; 73.2 ± 6.6 kg; 177.8 ± 7.8 cm	Competitive
Fernández-Baeza et al. (2022) (13)	Assess effect of individual TMG-based training on knee flexors.	*N* = 34 professional players	24.3 y; 74 kg; 179 cm	Competitive
Fabok et al. (2019) (3)	Quantify neuromuscular properties of BF and bilateral dimorphism.	*N* = 54 professionals	23.0 ± 4.4 y; 81.2 ± 15.1 kg; 182.6 ± 15.1 cm	Competitive
Padrón-Cabo et al. (2023) (26)	Assess maturation effects on RF and BF in elite youth players.	*N* = 121 elite youth players	14.9 ± 1.8 y; 60.6 ± 11.7 kg; 167.4 ± 10.4 cm	Competitive
Rey et al. (2012) (15)	Analyse differences in contractile response by playing position.	*N* = 78 professionals	26.6 ± 4.4 y; 75.8 ± 5.3 kg; 179.2 ± 5.3 cm	Competitive
Fernández-Baeza et al. (2022) (27)	Monitor seasonal changes in BF and ST neuromuscular properties.	*N* = 27 professional players	25.0 ± 4.5 y; 75.5 ± 7.7 kg; 179 ± 6 cm	Pre-season & competitive
Alentorn-Geli et al. (2015) (28)	Assess TMG properties as risk factors for ACL injury.	*N* = 78 (40 ACL injured, 38 controls)	Injured: 22.3 ± 6.8 y; 71.7 ± 7.7 kg; 175 ± 10 cm/Controls: 21.1 ± 4.9 y; 71.5 ± 10 kg; 175 ± 10 cm	Not described
García-García et al. (2017) (10)	Determine preseason muscle profile, symmetry and positional role differences.	*N* = 16 professionals (various positions)	28.2 ± 4.4 y; 74.5 ± 4.3 kg; 178.8 ± 6 cm	Pre-season
Alhowimel et al. (2022) (29)	Generate normative neuromuscular data of knee flexors/extensors.	*N* = 83 professionals	24.9 ± 4.1 y; 70.5 ± 9.1 kg; 175.3 ± 13.3 cm	Pre-season

Kg, kilograms; cm, centimetres.

**Table 3:** Description of the muscles and TMG variables.

Author(s), year	Muscles (TMG)	TMG variables
Rey et al. (2020) (23)	BF, RF	Dm, Tc, Vc
Álvarez-Díaz et al. (2016) (24)	VM, VL, RF, ST, BF, GM, GL	Dm
Fernández-Baeza and González-Millán (2020) (25)	RF, VM, BF, ST	Dm, Tc, Ts, Tr
Fernández-Baeza et al. (2022) (13)	BF, ST	Dm, Tc
Fabok et al. (2019) (3)	BF	Dm, Tc, Td, Ts, Tr, Vc
Padrón-Cabo et al. (2023) (26)	RF, BF	Dm, Td, Tc, Vc (Dm/Tc + Td)
Rey et al. (2012) (15)	BF, RF	Dm, Ts, Tc, Td, Tr
Fernández-Baeza et al. (2022) (27)	BF, ST	Tc, Dm, Td
Alentorn-Geli et al. (2015) (28)	VM, VL, RF, ST, BF	Dm, Ts, Tc, Td, Tr
García-García et al. (2017) (10)	VM, VL, RF, BF	Dm, Ts, Tc, Td, Tr, Vc
Alhowimel et al. (2022) (29)	RF, VL, VM, BF	Dm, Ts, Tc, Td, Tr

TMG, tensiomyography; BF, biceps femoris; RF, rectus femoris; VM, vastus medialis; VL, vastus lateralis; ST, semitendinosus; GM, gastrocnemius medialis; GL, gastrocnemius lateralis; Dm, maximum displacement; Tc, contraction time; Ts, duration; Tr, relaxation time; Td, response time; Vc, contraction velocity = Dm/(Tc + Td).

Secondly, the methodological quality of the studies and the quality of the reliability and measurement error properties of the TMG were evaluated. Finally, a synthesis of the best evidence was made.

### 2.5 Methodological evaluation of quality

One author independently evaluated the methodological quality using the quality assessment tool for cross-sectional and cohort observational studies, published by the National Heart, Lung, and Blood Institute (NHLBI). This tool consists of 14 questions, each of which could be marked with Yes, No or Not informed/Not applicable. A score of 1 is assigned to Yes, and a score of 0 to all other answers. The total score is the number of affirmative answers. For the final qualitative evaluation, scores above 12 were considered good, those below 9 were considered weak, and those between 9 and 12 were rated as fair (30).

## 3 Results

### 3.1 Selection and characteristics of the studies

A total of 489 articles were identified in the initial search of the databases, and 37 additional articles were found through other sources (Figure 1). After the elimination of duplicates, 462 articles remained. After reading the titles and abstracts, 365 records were excluded. The full texts of 24 articles were evaluated for eligibility. Each article was coded according to the characteristics of the study and its objectives, parameters derived from the TMG, musculature evaluated, and results obtained (Table 2, Table 3 and Table 4). Finally, 11 articles, published from 2012 to 2024, were included in this systematic review with a total of 570 elite male soccer players with a weighted mean age of 23.8 ± 4.4 years. The weighted average was calculated by taking the arithmetic mean of all values.

**Table 4:** Study results.

Section	Authors	Main findings
Contractile properties and Neuromuscular state	Rey et al. (23)	No correlation between Tc and training load; no progression changes in Dm, Tc, Vc of RF and BF during microcycle.
Contractile properties and Neuromuscular state	García-García et al. (31), (10)	Tc lower at preseason start vs. in-season; BF showed higher values in-season; Dm lower preseason vs. season across all muscles; Td remained similar.
Contractile properties and Neuromuscular state	Fernández-Baeza et al. (27)	Preseason vs. competitive season: ST Dm worsened and increased, indicating slower muscle with time.
Contractile properties and Neuromuscular state	Fernández-Baeza et al. (13)	BF and ST, despite being synergists, showed different Tc and tone patterns.
Contractile properties and Neuromuscular state	Padrón-Cabo et al. (26)	Examined maturation effects (RF, BF). No significant differences in Tc, Td, Vc across PHV stages. Similar Dm values across maturation stages, but lower Dm and higher Tc vs. professionals.
Contractile properties and Neuromuscular state	Fernández-Baeza and González-Millán (25)	Professionals showed higher Ts (except ST), significant in BF, RF, VL, VM. Tr differences in VL and BF. Dm differences significant in RF, with professionals having lower values. VL and VM identified as most explosive.
Muscle asymmetry	Fabok et al. (3)	No bilateral dimorphism; altered distribution in Tr between dominant and non-dominant legs.
Muscle asymmetry	Alhowimel et al. (29)	Low lateral symmetry in BF; correct symmetry in flexors. Largest difference in VL (10.2%). RF Dm asymmetry noted.
Muscle asymmetry	Álvarez-Díaz et al. (24)	No influence of foot dominance on TMG parameters.
Muscle asymmetry	Alentorn-Geli et al. (28)	Greatest asymmetry in VM, lowest in BF.
Positional roles	Alhowimel et al. (29)	No effect of player position on TMG values.
Positional roles	Rey et al. (15)	Significant influence of playing position on Tc, Ts, Td of RF; midfielders had lower Ts in RF.
Positional roles	Fernández-Baeza and González-Millán (25)	Defenders had lower Dm in VM, VL, and BF than attackers.
Positional roles	García-García et al. (10)	Several differences in VM, RF, and BF depending on position; only Tr in BF and Ts in VM consistent across positions.
Injury risk	Alentorn-Geli et al. (28)	TMG parameters higher on uninjured side of ACL group.
Injury risk	Alentorn-Geli et al. (28)	ACL-injured players had higher Dm and Tc in healthy limb.
Dominant vs. non-dominant leg	Fernández-Baeza and González-Millán (25)	No differences in left-footed players. Right-footed players: higher Ts in VM and lower Tc in BF of dominant leg.
Dominant vs. non-dominant leg	Álvarez-Díaz et al. (24)	TMG characteristics not influenced by leg dominance.

TMG, tensiomyography; PHV, peak height velocity; ACL, anterior cruciate ligament; BF, biceps femoris; RF, rectus femoris; VM, vastus medialis; VL, vastus lateralis; ST, semitendinosus; GM, gastrocnemius medialis; GL, gastrocnemius lateralis; Dm, maximum displacement; Tc, contraction time; Ts, duration; Tr, relaxation time; Td, response time; Vc, contraction velocity = Dm/(Tc + Td).

The minimum sample size per study was 16 (10), while the maximum was 121 (26).

The muscles measured were rectus femoris (RF), vastus lateralis (VL), vastus medialis (VM), biceps femoris (BF), and semitendinosus (ST).

### 3.2 Data extraction and methodology

Information and data extraction was carried out independently by one reviewer (JL) and verified by a second reviewer (AB) using an extraction sheet.

﻿The authors evaluated the quality of the results, screening based on the title and abstracts according to the inclusion criteria. The full texts were then selected by the same authors.

The average NHLBI was 10, with values ranging from 9 to 12, which suggests that in general the included studies were of fair quality (30).

### 3.3 Study types and measurement properties of the included studies

The study types and measurement properties of the included studies are summarized in Table 2 and Table 3.

Rey et al. (23) examined changes in the muscle contractile properties of the BF and RF throughout a training micro cycle in professional soccer players, during the season, using a repeated measures design. They measured the values of TMG Dm, Tc, and Vc.

Álvarez-Diaz et al. (24) compared the neuromuscular characteristics between the dominant and non-dominant lower limb using TMG in male soccer players in the competitive period through a cross-sectional, intergroup, and comparative study of a group of 38 players made up of 12 forwards, 8 midfielders, and 18 defenders, of whom 6 were left-handed and 32 were right-handed. The muscles measured were VM, VL, RF, ST, BF, and the TMG variable Dm.

The contractile properties of the superficial knee flexor and extensor muscles joint of both legs were compared by Fernández-Baeza and González-Millán (25) in professional and amateur soccer players, with the aim of showing the differences between the two groups. The muscles evaluated were RF, VM, VL, BF, and ST, and the TMG variables Dm, Tc, Ts, Tr.

Fernández-Baeza et al. (13) analyzed the effect of an individual training program based on the results of tensiomyography on the contractile properties of the knee flexor muscle in soccer players, in a comparative experimental study on BF and ST through the measurement of Dm and Tc.

The objective of Fabok et al. (3) was to define the quantitative neuromuscular value of the characteristics of the BF muscle as a flexor of the knee joint, and to compare all the characteristics of TMG-BF with the bilateral dimorphism of the variables examined. The Dm, Tc, Td, Ts, Tr, and Vc of the BF, were measured in the competitive period.

Padrón-Cabo et al. (26) carried out a comparative cross-sectional study during the competitive period to determine the effects of maturation on the contractile properties of the RF and BF muscles with the aim of providing reference values for elite youth soccer players. They measured Dm, Td, Tc and Vc.

A descriptive study by Rey et al. (15) analyzed the differences in muscle response and the mechanical characteristics of the main lower limb muscles in a large group of Spanish soccer players according to playing position. BF and RF were measured using the variables Dm, Ts, Tc, Td, and Tr.

In a comparative study, Fernández-Baeza et al. (27) monitored seasonal changes in the mechanical and neuromuscular characteristics of the knee flexor muscles, BF and ST, during the preseason and the competitive period, measuring Tc, Dm and Td.

The objective of Alentorn-Geli et al. (28) was to investigate the role of the mechanical contractile properties of the VM, VL, RF, ST and BF muscles, as risk factors for anterior cruciate ligament (ACL) injury in a comparative, cross-sectional, controlled and intergroup study. They evaluated a group of 40 injured soccer players (ACL group) and 38 healthy ones (Control group) by measuring the variables Dm, Ts, Tc, Td, and Tr.

García-García et al. (10) determined the mechanical and neuromuscular muscle profile of the knee flexo-extensor muscles (VM, VL, RF and BF) at the beginning of the preseason, and calculated the percentages of symmetry, according to the positional role of the players using Dm, Ts, Tc Td, Tr and Vc.

Finally, Alhowimel et al. (29) generated normative data on the mechanical and neuromuscular profile of the RF, VL, VM and BF knee extensor and flexor muscles in a cross-sectional study at the start of the preseason, measuring Dm, Ts, Tc Td and Tr.

## 4 Discussion

The main objective of the review was to analyze the usefulness of the TMG device as a control tool for evaluating the neuromuscular characteristics of the knee flexor and extensor muscles in elite male soccer players. A total of eleven eligible studies were identified that investigated the contractile parameters of these muscles using TMG.

### 4.1 Protocol of obtaining measurements using TMG

The measurement conditions were the same in all the selected studies, with the subjects in static and relaxed resting conditions in a supine position for the VM, VL and RF, and in a prone position for the ST and BF muscles. In the supine position, the knee was fixed at angles of 60°, 120°, 140°, 150° and 165° (180° being full knee extension).

A wedge-shaped foam cushion designed for this purpose was used in many of them.

The articles listed in Table 3 used the same digital displacement transducer for measurement (GK 40, Panoptik Ltd., Ljubljana, Slovenia), except for García-García et al. (10); Fernández-Baeza et al. (13) and Fernández-Baeza et al. (27), who used the GK 30, Panoptik Ltd., Ljubljana, Slovenia. Fernández-Baeza and González-Millán (25) did not report on the equipment used, and Alhowimel et al. (29) used TMG-S2.

All the studies located the position of the sensor tip (that is, the most prominent area of the muscle belly), using the same (or similar) anatomical guide, always placing it perpendicular to the muscle belly.

The TMG-S2 electro-stimulator (EMF- Furlan Co. & Ltd., Ljubljana, Slovenia) was used most (five studies), followed by the TMG-100 (TMG-BMC Ltd., Ljubljana, Slovenia) and TMG-S1 (Furlan Co. & Ltd., Ljubljana, Slovenia) with three uses each. Only Fernández-Baeza and González-Millán (25) did not provide information.

All the studies applied rest time intervals of 10 or 15 s between successive evaluations, except for four that did not detail this data (Alentorn-Geli et al. (28); Álvarez-Diaz et al. (24); Fernández-Baeza and González-Millán (25) and Alhowimel et al. (29).

In four studies electrodes were used from Cefar-Compex Medical AB Co., Ltd, Malmö, Sweden, TMG-BMC Ltd., Ljubljana, Slovenia in three, two from Compex Medical SA, Ecublens, Switzerland, and only in two of them was this not reported. Both electrodes (5 × 5 cm) were placed symmetrically to the sensor; the positive electrode (anode) was placed proximally and the negative electrode (cathode) distally, 50–60 mm from the digital sensor. In some cases, the location of the sensor and electrodes was marked with a semi-permanent marker.

Almost all the studies used an initial stimulus with a duration of 1 ms of a monophasic pulse of between 20 and 50 milliamperes (mA), with progressive increases of 5–10 mA, until there was a new increase in the Dm, or until reaching the maximum electrical power provided by the equipment (100–110 mA). In Rey's study (15) they used varied stimuli (50, 75 and 100 mA), and only Alhowimel et al. (29) did not report data.

The number of evaluators in all cases was one or two, always being people experienced in taking measurements. In the cases in which two evaluators were used, one was responsible for monitoring the intensity and frequency of the applied stimulus, while the other supervised the location of the sensor and the recording of the leg being evaluated.

The curves with the highest maximum radial displacement were selected for the results. If they were measured twice, the first measurement was used to ensure the correct functioning of the TMG, and the second was used as the definitive value.

### 4.2 Populations evaluated and objectives of the original projects

Populations evaluated and objectives of the original projects are summarized in Table 4.

#### 4.2.1 Contractile properties and neuromuscular state

Rey et al. (23) found no correlation between absolute changes in Tc and training load variables. Nor did they find changes in the progression of muscle stiffness in Dm, Tc and Vc of the RF and BF caused by the training sessions during the micro cycle, possibly due to the recovery of the neuromuscular state of professional soccer players between training sessions throughout the micro cycle (23).

García-García et al. (31) found that the Tc values at the start of the preseason were lower than during the season and García-García et al. (10), using the same methodology, found, however, that the BF knee flexor muscle had higher values. The Dm values at the start of the preseason were lower than during the season in all the muscles evaluated and the Td values were like those obtained during the season. All the results obtained by these authors support the hypothesis that the Tc, Td and Dm variables of professional soccer players evolve throughout the season, as already suggested by García-García et al. (31).

Fernández-Baeza et al. (27) compared the data from the preseason and the competitive season, with the initial ST Dm values being worse and higher as the season progressed. This indicates that the ST muscle became a slower muscle as the season progressed. On the contrary, the BF values improved throughout the season, as did its explosiveness. The BF also improved its muscle tone throughout the season based on the Dm. These data from Fernández-Baeza et al. (27) do not coincide with the statement by García-García et al. (31) who indicated that the BF muscle slightly worsened its Tc and Dm during the season. As for the ST muscle, its Dm worsened, increasing during the season, which means that as the season progressed, the ST muscle tone worsened, coinciding with García-García et al. (31). The authors suggest that these differences may be attributed to the absence of a strength training programme incorporating analytical exercises targeting the semitendinosus, aimed at balancing the intermuscular coordination elicited by training and competition between the BF and the ST.

In another study, Fernández-Baeza et al. (13) they argued that the BF and ST muscles, despite being synergists and sharing the proximal tendon, present different Tc and muscle tone patterns. They observed that during sprinting there is greater activation of the BF compared to the ST, which may be the explanation for why at the beginning of the competitive period, when sprints increase in frequency, the differences between these muscles also increase. Lack of coordination between the two muscles would increase the risk of injury, with it being more likely to occur in the BF (32).

Padrón-Cabo et al. (26) conducted a study to determine the effects of maturation on the contractile properties of RF and BF muscles in elite youth soccer players. They measured Dm, Tm, Tc and Vc, pre, mid and post peak height velocity (PHV). The data did not reveal significant differences in the variables Tc, Td and Vc in the RF and BF between Pre, mid and post in PHV. In this sense, contrary to what was expected, there were similar Dm values for the RF and BF muscle groups according to the stage of maturation in young soccer players. However, they showed a lower Dm and a higher Tc than adult professional soccer players, possibly due to greater muscle stiffness and a higher proportion of fast fibers (15).

The authors recommend caution when interpreting these results, as the absence of differences between stages of maturation may be related to chronic adaptations derived from the systematic exposure of elite youth football players to training in a highly competitive environment, as well as to the sample composition, which included a low representation of players in the Pre- and Mid-PHV stages.

Comparing the contractile properties of the superficial knee flexor and extensor muscles in professional and amateur soccer players, Fernández-Baeza and González-Millán (25) showed that in the Ts variable, they had higher data in professionals than in amateurs in all muscles (except in the ST) and significantly in BF, RF, VL and left and right VM. Also, with Tr, significant differences were observed between them in VL of both legs, and in right BF. Regarding the Dm variable, related to strength, they saw significant differences in RF of both legs in these groups.

Finally, in the Tc variable in the left VL, they found higher values in professionals. In this study, both in professionals and amateurs, the muscles with the lowest Tc (more explosive muscles) were the VL and VM. These data coincide with the results obtained by García et al. (31).

Fernández-Baeza and González-Millán (25) also reported that the slowest muscle in both groups was the ST, coinciding with Álvarez-Díaz et al. (24), although the former obtained higher (slower) data probably due to the average age of the sample, which was higher in their study (25).

With regard to Dm, significant differences were observed between groups, with lower values in professionals compared to amateurs.

According to Fernández-Baeza and González-Millán (25), the greater number of training days and the higher competitive intensity in professional players are likely to result in increased muscle stiffness, which may explain these differences in the outcomes.

#### 4.2.2 Muscle asymmetry

Fabok et al. (3) demonstrated that there is no bilateral dimorphism in the Serbian professional soccer players examined. However, there is a slightly altered and less homogeneous distribution in the Tr variable of dominant and non-dominant legs, due to a possible chronicity of the phenomenon of fatigue, which is identified by the increasing value of this variable (3).

On the other hand, Alhowimel et al. (29) during the start of the preseason, in professional soccer players of the Saudi Arabian Premier League, highlighted a low lateral symmetry in the knee extensor muscle BF, on the contrary, all the flexor muscles demonstrated a correct symmetry. There was a minimal difference in knee extensor asymmetry (2.5%), however, the most considerable difference was observed in the VL (10.2%). A difference between right and left neuromuscular measurements was observed only in RF Dm.

On the contrary, the parameters were not affected by foot dominance (24). It should be acknowledged that this study was conducted during the competitive season, which may have introduced a certain degree of fatigue, competition and opponents, and consequently influenced the results (13).

Alentorn-Geli et al. (28) find the greatest asymmetry was observed in the VM muscle and the lowest in the BF. The potential influence of ACL injury on the contralateral healthy side may not be negligible with regard to the results.

Several studies have associated muscle asymmetry with injury prevalence and athletic performance. Beyond its predictive value for injuries, asymmetry may also support clinical decision-making regarding the return to play of football players following injury (33).

Álvarez-Díaz et al. (24) acknowledge certain potential limitations in the acquisition of the results, as the measurements were conducted under static and resting conditions, which differ substantially from on-field characteristics.

#### 4.2.3 Variations according to playeŕs positional roles

The result for Alhowimel et al. (29), suggests that the player's positions do not affect the TMG values. However, these results contradict the data reported for Spanish elite soccer players, which suggests a significant influence of player position on Tc, Ts, and Td of RF (15). According to these authors, midfielders had significantly lower Ts values for RF than the other positions. It should be noted that the latter study was conducted during the season, which may introduce a certain level of fatigue and influence the results (10).

Fernández-Baeza and González-Millán (25) did find significant differences in terms of the positional role in the Dm, this being lower in defenders than in attackers in the left VM, right VL, left VL and left BF respectively. This indicates more powerful muscles in defenders. One possible explanation from the authors would be the different demands of training and competition depending on the playing position in football, as defenders have a higher level of performance in vertical jump and speed than other positional roles (25).

García-García et al. (10) also observed several significant differences in the TMG variables for VM, RF and BF depending on the positional role of the players. However, only Tr in the BF and Ts in the VM differed in all the playing positions considered. Several significant differences were observed in the TMG variables for VM, RF and BF depending on the positional role of the players. However, only the Tr in the BF and the Ts of the VM differed in all the playing positions considered (10).

The discrepancies between these results and those reported by Alhowimel et al. (29), may be attributed to differences in data collection procedures, the timing of the assessments within the season, and/or the competitive level of the players involved (10).

#### 4.2.4 Predictor of injury risk

Alentorn-Geli et al. (28) evaluated a cohort of 40 injured football players (ACL group) and 38 healthy players (control group), reporting the following findings: the vast majority of TMG parameters were higher on the non-injured side of the ACL-injured individuals compared with the control group; the values of Tr-VL, Tc-RF, Ts-RF, Tr-RF, and Dm-BF were significantly higher on the contralateral uninjured side than in controls; the quadriceps muscles exhibited greater significant differences than the hamstrings; and the RF was the muscle in which the most pronounced intergroup differences were observed.

They concluded that the alterations found in the BF may increase the risk of ACL injury, as the ability of the hamstrings to counteract anterior tibial rotation would be reduced due to a decrease in fatigue resistance and muscle stiffness or tone. Therefore, players would have less effective anterior cruciate ligament agonists (hamstrings). The altered parameters in the VL and RF may indicate an altered muscle contraction between the quadriceps and hamstrings, which may also increase the risk of ACL injury (28).

Also, in a previous study Alentorn-Geli et al. (28) reported that soccer players with anterior cruciate ligament injuries had higher Dm and Tc in the healthy limb than non-injured players. Therefore, the joint contraction of knee flexors and extensors could be related to their functional balance, which could be a key predictor of injury risk (33).

#### 4.2.5 Differences between dominant and non-dominant leg

Fernández-Baeza and González-Millán (25) found no significant differences between professional and amateur football players when comparing the dominant and non-dominant legs in left-footed players. However, in right-footed players, differences were observed in the Ts of the vastus medialis (VM), which was higher in the dominant leg, and in the Tc of the biceps femoris (BF), which was lower in the dominant leg.

Significant differences in the Tc of the BF were also reported in right-footed players, with lower values in the dominant leg, a finding that could be explained by greater explosiveness. In their study, Álvarez-Díaz et al. (24) concluded that the TMG characteristics of the main lower-limb muscles were not influenced by side dominance in male football players, a result consistent with those reported by Alentorn-Geli et al. (28) and García-García et al. (10).

### 4.3 Future research and perspectives

Despite its benefits, further research is required to fully understand its potential and limitations.

The differences observed between the three knee extensors and the biceps femoris suggest the need to monitor the hamstring-to-quadriceps ratio in football players throughout the season, with TMG being a useful tool for this purpose.

With regard to asymmetry values, additional investigation is still needed to precisely define which symmetry percentages represent reliable indicators of muscular asymmetry, whether lateral or functional.

Nevertheless, future studies are required following the same methodologies in the use of TMG in different professional teams, in order to concisely identify the baseline neuromuscular values of professional football players, both male and female.

## 5 Conclusions

TMG is a non-invasive and efficient method that does not interfere with athletes' daily routines. It requires a relatively simple set-up, and the devices are portable and relatively cost-effective in relation to the outcomes obtained, making them applicable in both clinical and sporting contexts. Moreover, it represents a valuable alternative to invasive techniques such as muscle biopsy.

TMG also constitutes a valuable assessment tool in football, enabling the monitoring of neuromuscular status throughout the season, the evaluation and supervision of players in terms of muscle injury risk and recovery processes, and the determination of their readiness to return to competition. Additionally, it provides information not only on individual responses to training, but also on neuromuscular and functional fatigue, as well as on lateral asymmetries.

The neuromuscular profile derived from TMG may assist coaches and medical staff in identifying baseline characteristics relevant to training planning and programming. Furthermore, during the competitive period, the results may contribute to assessing the specific effects of training on the muscle itself and to adapting training sessions accordingly.

## Data Availability

The original contributions presented in the study are included in the article/Supplementary Material, further inquiries can be directed to the corresponding author.

## References

[1] Loturco I Pereira LA Kobal R Kitamura K Ramírez-Campillo R Zanetti V Muscle contraction velocity: a suitable approach to analyze the functional adaptations in elite soccer players. J Sports Sci Med. (2016) 15. Available online at: http://www.jssm.org 27803627 PMC4974861

[2] Barnes C Archer DT Hogg B Bush M Bradley PS. The evolution of physical and technical performance parameters in the English premier league. Int J Sports Med. (2014) 35(13):1095–100. 10.1055/s-0034-1375695 25009969

[3] Fabok M Leontijević B Tomić L Dopsaj M. Neuromuscular characteristic of Biceps femoris muscle in the top Serbian soccer players measured by tensiomyography method: quantitative model. Facta Univ Ser Phys Educ Sport. (2019) 17(2):167. 10.22190/fupes190530018f

[4] Arnason A Sigurdsson SB Gudmundsson A Holme I Engebretsen L Bahr R. Risk factors for injuries in football. Am J Sports Med. (2004) 32(SUPPL. 1). 10.1177/0363546503258912 14754854

[5] Woods C Hawkins RD Maltby S Hulse M Thomas A Hodson A. The football association medical research programme: an audit of injuries in professional football—analysis of hamstring injuries. Br J Sports Med. (2004) 38(1):36–41. 10.1136/bjsm.2002.002352 14751943 PMC1724733

[6] García-Manso JM Rodríguez-Matoso D Sarmiento S De Saa Y Vaamonde D Rodríguez-Ruiz D La tensiomiografía como herramienta de evaluación muscular en el deporte. Rev Andal Med Deporte. (2010) 3(3):98–102. https://www.elsevier.es/ramd

[7] McHugh MP Connolly DA Eston RG Kremenic IJ Nicholas SJ Gleim GW. The role of passive muscle stiffness in symptoms of exercise-induced muscle damage. Am J Sports Med. (1999) 27(5):594–9. 10.1177/03635465990270050801 10496575

[8] Watsford ML Murphy AJ McLachlan KA Bryant AL Cameron ML Crossley KM A prospective study of the relationship between lower body stiffness and hamstring injury in professional Australian rules footballers. Am J Sports Med. (2010) 38(10):2058–64. 10.1177/0363546510370197 20595555

[9] Palmer TB Thompson BJ Hawkey MJ Conchola EC Adams BM Akehi K The influence of athletic Status on the passive properties of the muscle-tendon unit and traditional performance measures in division in female soccer players and nonathlete controls. J Strength Cond Res. (2014) 28(7):2026–34. 10.1519/JSC.0000000000000332 24276302

[10] García-García O Serrano-Gomez V Hernandez-Mendo A Morales-Sánchez V. Baseline mechanical and neuromuscular profile of knee extensor and flexor muscles in professional soccer players at the start of the Pre-season. J Hum Kinet. (2017) 58(1):23–34. 10.1515/hukin-2017-0066 28828075 PMC5548152

[11] Macgregor LJ Hunter AM Orizio C Fairweather MM Ditroilo M. Assessment of skeletal muscle contractile properties by radial displacement: the case for tensiomyography. Sports Med. (2018) 48:1607–20. 10.1007/s40279-018-0912-6 29605838 PMC5999145

[12] Čular D Babić M Zubac D Kezić A Macan I Peyré-Tartaruga LA Tensiomyography: from muscle assessment to talent identification tool. Front Physiol. (2023) 14:1163078. 10.3389/fphys.2023.1163078 37435303 PMC10330706

[13] Fernández-Baeza D Diaz-Ureña G González-Millán C. Effect of an individualised training programme on hamstrings and change direction based on tensiomyography in football players. Appl Sci. (2022) 12(21). 10.3390/app122110908

[14] Carrasco L Sañudo B De Hoyo M Pradas F Da Silva M. Effectiveness of low-frequency vibration recovery method on blood lactate removal, muscle contractile properties and on time to exhaustion during cycling at VO 2max power output. Eur J Appl Physiol. (2011) 111(9):2271–79. 10.1007/s00421-011-1848-9 21327798

[15] Rey E Lago-Penas C Lago-Ballesteros J. Tensiomyography of selected lower-limb muscles in professional soccer players. J Electromyogr Kinesiol. (2012) 22(6):866–72. 10.1016/j.jelekin.2012.06.003 22776612

[16] Tous-Fajardo J Moras G Rodríguez-Jiménez S Usach R Doutres DM Maffiuletti NA. Inter-rater reliability of muscle contractile property measurements using non-invasive tensiomyography. J Electromyogr Kinesiol. (2010) 20(4):761–6. 10.1016/j.jelekin.2010.02.008 20236839

[17] García-Manso JM Rodríguez-Ruiz D Rodríguez-Matoso D De Saa Y Sarmiento S Quiroga M. Assessment of muscle fatigue after an ultra-endurance triathlon using tensiomyography (TMG). J Sports Sci. (2011) 29(6):619–25. 10.1080/02640414.2010.548822 21391085

[18] Turner AN Stewart PF. Strength and conditioning for soccer players. Strength Cond J. (2014) 36(4):1–13. 10.1519/SSC.0000000000000054

[19] Page MJ McKenzie JE Bossuyt PM Boutron I Hoffmann TC Mulrow CD The PRISMA 2020 statement: an updated guideline for reporting systematic reviews. Br Med J. (2021) 372. 10.1136/bmj.n71 PMC8005924 33782057

[20] Slobogean GP Verma A Giustini D Slobogean BL Mulpuri K. MEDLINE, EMBASE, and cochrane index most primary studies but not abstracts included in orthopedic meta-analyses. J Clin Epidemiol. (2009) 62(12):1261–7. 10.1016/j.jclinepi.2009.01.013 19364634

[21] Prinsen CA Mokkink LB Bouter LM Alonso J Patrick DL De Vet HC COSMIN guideline for systematic reviews of patient-reported outcome measures. Qual Life Res. (2018) 27(5):1147–57. 10.1007/s11136-018-1798-3 29435801 PMC5891568

[22] Leeflang MMG Deeks JJ Takwoingi Y Macaskill P. Cochrane diagnostic test accuracy reviews. Syst Rev. (2013) 2:82. 10.1186/2046-4053-2-82 24099098 PMC3851548

[23] Rey E Corredoira F Costa PB Pérez-Ferreirós A Fernández-Villarino M. Acute effects of training load on contractile properties during a competitive micro cycle in elite soccer players. Biol Sport. (2020) 37(2):157–63. 10.5114/BIOLSPORT.2020.93041 32508383 PMC7249794

[24] Alvarez-Diaz P Alentorn-Geli E Ramon S Marin M Steinbacher G Rius M Comparison of tensiomyographic neuromuscular characteristics between muscles of the dominant and non-dominant lower extremity in male soccer players. Knee Surg Sports Traumatol Arthrosc. (2016) 24(7):2259–63. 10.1007/s00167-014-3298-5 25236679

[25] Fernández-Baeza D González-Millán C. (Descriptive study of the contracting properties of the flexo-extension muscles of knee in professional and affected football players). Estudio descriptivo de las propiedades contráctiles de los músculos flexo-extensores de rodilla en futbolistas profesionales. J Sport Health Res. (2020) 12(3):458–71.

[26] Padrón-Cabo A Corredoira FJ Lorenzo-Martínez M González-Víllora S Rey E. Tensiomyographic assessment of Contractile properties in elite youth soccer players according to maturity status. J Hum Kinet. (2023) 87:71–80. 10.5114/jhk/161571 37229402 PMC10203836

[27] Fernández-Baeza D Diaz-Ureña G González-Millán C. Differences in the contractile properties of the biceps femoris and semitendinosus muscles throughout a season in professional soccer players. J Hum Kinet. (2022) 84(1):74–81. 10.2478/hukin-2022-0088 36457475 PMC9679184

[28] Alentorn-Geli E Alvarez-Diaz P Ramon S Marin M Steinbacher G Boffa JJ Assessment of neuromuscular risk factors for anterior cruciate ligament injury through tensiomyography in male soccer players. Knee Surg Sports Traumatol Arthrosc. (2015) 23(9):2508–13. 10.1007/s00167-014-3018-1 24807228

[29] Alhowimel A Faris A Yaaqoub K Hana A Othman A. The neuromuscular profile of knee extensor and flexor muscles in professional soccer players in the Saudi premier league. Sport TK. (2022) 11(2). https://revistas.um.es/sportk

[30] Pus K Paravlic AH Šimunič B. The use of tensiomyography in older adults: a systematic review. Front Physiol. (2023) 14:1213993. 10.3389/fphys.2023.1213993 37398907 PMC10311920

[31] García-García O Serrano-Gomez V Hernandez-Mendo A Tapia-Flores A. Assessment of the in-season changes in mechanical and neuromuscular characteristics in professional soccer players. J Sports Med Phys Fitness. (2015) 56(6):714–23. 26393480

[32] Wilmes E De Ruiter CJ Bastiaansen BJ Goedhart EA Brink MS Van der Helm FC Associations between hamstring fatigue and sprint kinematics during a simulated football (soccer) match. Med Sci Sports Exercise. (2021) 53(12):2586. 10.1249/MSS.0000000000002753 PMC8594518 34265817

[33] Wang J Fu W. Asymmetry between the dominant and non-dominant legs in the lower limb biomechanics during single-leg landings in females. Adv Mech Eng. (2019) 11(5):1687814019849794. 10.1177/1687814019849794

[34] Martín-Rodríguez S Loturco I Hunter AM Rodríguez-Ruiz D Munguia-Izquierdo D. Reliability and measurement error of tensiomyography to assess mechanical muscle function: a systematic review. J Strength Cond Res. (2017) 31(12):3524–36. 10.1519/JSC.0000000000002250 28930871

